# Effect of an intensive tobacco cessation program on the smoker narrative: A content analysis and grounded theory

**DOI:** 10.18332/tid/183607

**Published:** 2024-02-15

**Authors:** Carlos Rábade-Castedo, Carlos Zamarrón-Sanz, Álvaro Hermida-Ameijeiras, Romina Abelleira-Paris, Ana Casal-Mouriño, Lucia Ferreiro-Fernández, Nuria Rodríguez-Núñez, Jorge Ricoy-Gabaldón, María Elena Toubes-Navarro, José Manuel Álvarez-Dobaño, Luis Valdés-Cuadrado

**Affiliations:** 1 Servicio de Neumología, Complejo Hospitalario Universitario de Santiago de Compostela, Universitario de Santiago de Compostela, Santiago de Compostela, España; 2 Instituto de Investigación Sanitaria de Santiago de Compostela (IDIS), Santiago de Compostela, España; 3 Servicio de Medicina Interna, Complejo Hospitalario Universitario de Santiago de Compostela, Universitario de Santiago de Compostela, Santiago de Compostela, España

**Keywords:** cessation, smoking, tobacco, dependence

## Abstract

**INTRODUCTION:**

The smoker's narrative during smoking quitting provides insight into aspects not fully explored in daily clinical practice. The aim of the study was to analyze the smoker narrative using two types of methodologies: content analysis and grounded theory, before and after smoking cessation intervention, provided to the smoker in a specialized Smoking Cessation Unit accredited by the Spanish Society of Pneumology and Thoracic Surgery.

**METHODS:**

A prospective observational study of current smokers included in a tobacco cessation program between 2017 and 2020 was conducted at the Smoking Cessation Unit of Santiago de Compostela Health Area, Spain. Routine clinical variables and patient narrative data were collected. A descriptive analysis of the sample, the content of the textual corpus, and a grounded theory were performed in semi-structured interviews at baseline and at follow-up at 6 months.

**RESULTS:**

A total of 116 patients were included (mean age 55.6 ± 10.6 years; 56.9% male; mean nicotine dependence score 5.7 ± 1.6). Quantitative analysis of the narrative shows that the most frequent phrases and words are associated with smoking, nicotine craving, and predisposition for smoking cessation. After the intervention, phrases related to the manifestation of abstinence, response to pharmacological treatment, and self-perception of smoking cessation were predominant. In the qualitative analysis, the most frequent categories in the smoker's textual corpus were dependence, motivation, and emotionality, which decreased after the intervention (11.4%, 21.4%, and 9.9%, respectively) accompanied by increased satisfaction (19.2%) and the manifestation of abstinence (21.5%).

**CONCLUSIONS:**

Motivation, nicotine dependence, and sensitivity to emotions are all closely intertwined in the current smoker narrative and can be modified as a consequence of treatment.

## INTRODUCTION

Smoking is an addictive and chronic disease that has a high impact in terms of morbidity, mortality, and quality of life. Therefore, it requires different levels of healthcare approaches (community-oriented and specialized medical care). Smokers are cared for at the basic level of care by primary care physicians, dentists, and community nurses. This care is characterized by proximity, accessibility, and continuity of care with the smoker. However, they are less intensive and less successful interventions. The basic level of smoking cessation care is characterized by an intervention based on the 5As (Ask, Advise, Assess, Assist, and Arrange). It lasts 3 to 5 minutes, with a maximum of 4 visits and a follow-up of 6 months. Smoking cessation interventions in specialized care are performed by professionals with high competence in smoking cessation. The smokers are treated in specialized smoking quitting centers. Despite being aimed at a population of smokers with greater difficulties in quitting, they have been proven to be both effective and cost-effective. All of them are based on the diagnosis, treatment, and follow-up of the smoker. The intervention consists of psychological counseling and pharmacological treatment. The combination of both actions triples the chances of quitting, and almost 50% succeed^1-5^.

However, a high percentage are not able to remain smoke-free. This could be attributed to the fact that the questionnaires and diagnostic tests on smoking do not include individual aspects of the smoker that would allow us to improve the effectiveness of treatment and adapt strategies for each patient. Moreover, they only reflect part of the reality of smoking. These tests are susceptible to the lack of sincerity in the smoker’s answers, constituting a rigid and identical assistance model for all. On the other hand, we often express the results of a smoking cessation program in terms of efficacy rates without exploring other qualities that change during the cessation process, such as sensitivity to emotions, the need for nicotine, or the degree of satisfaction. These aspects may be key to ensuring the maintenance of abstinence or negativity to a new serious quit attempt in those smokers who relapse. A recent study shows that the most frequent causes of relapse are: 1) positive and negative emotions; 2) withdrawal syndrome, and craving motivated by nicotine dependence^6-8^.

The smoking process is complex and encompasses multiple aspects (emotions, external stimuli, experiences, accompaniment, social relationships, damage caused by smoking, and the impact of previous quit attempts). The way the smokers express themselves in their narrative will allow us to capture the patient’s experience and facilitate the visualization of the different dimensions of the problem, as well as possible solutions. The narration of the smoker’s experiences with smoking is conducted with a semi-structured interview, and its analysis uses qualitative methodology, as has been recently reported^9,10^. However, exclusively qualitative analyses are inaccurate and subjective. To correct these limitations, a technique that incorporates qualitative and quantitative methodology (content analysis) is used. That is, it makes it possible to determine the frequency of phrases and words in the text with meaning, the frequency of categories or codes in the text, and the relationship between categories or codes, which leads to the formulation of the grounded theory, as well as to identify the smoker’s qualities during the different phases of the smoking cessation process, evaluate the degree of drug satisfaction regarding nicotine dependence, or the characteristics of the smoker in specific populations^11-16^. Despite these studies, their application continues to be scarce in this field.

The aim of the study was to analyze the smoker narrative using two types of methodologies: content analysis and grounded theory, before and after smoking cessation intervention, provided to the smoker in a specialized Smoking Cessation Unit accredited by the Spanish Society of Pneumology and Thoracic Surgery.

## METHODS

### Study design

This was an observational and prospective study of smokers who consecutively attended the Smoking Cessation Unit of the Clinic Hospital, Santiago de Compostela, Spain, between 2017 and 2020.

### Population

Patients of both sexes aged 18–70 years who were trying to quit smoking were included, regardless of whether they had had previous quit attempts, successful or unsuccessful. Those who did not agree to participate in the study or who did not wish to quit smoking, were excluded.

The methodology used for the main objectives is qualitative, so no formal justification of the sample size is necessary. Patients were included until data saturation was achieved, i.e. a level where no new codes were created, and the categories of analysis were not modified. To achieve the objectives, we took into account the number of patients attending the smoking consultation (150/year). Given the complexity of the study, the rate of acceptance to participate was low (32%/year).

### Study phases

The study consisted of three phases: 1) Care and general analysis, 2) Narrative, and 3) Content analysis.

*Care and general analysis phase*

Data collection was part of normal clinical care of patients in participating practices on a daily basis: filiation and anthropometric data, identification of the smoking cessation phase, number of previous quit attempts made and cause of relapse, nicotine dependence score (Fagerström test for nicotine dependence, and Glover-Nilsson Smoking Behavior Questionnaire), analysis of motivation and self-efficacy using the visual analog scale, and associated comorbidities. We used the Minnesota Tobacco Withdrawal Scale to determine the smoker’s abstinence syndrome during the smoking cessation process at baseline and at follow-up at 6 months. Carbon monoxide (ppm) in exhaled air was determined by co-oximetry.

*Narrative phase*

A semi-structured interview was conducted based on a model of open-ended questions about smoking that allowed the smoker to express himself/herself freely (Table 1). The narrative was collected, consisting of unstructured data at the smoker’s baseline visit and at follow-up at 6 months after starting smoking cessation. In other words, a corpus of clinical narratives before and after treatment was obtained. The textual corpus was compiled from a set of texts as a result of the patient’s clinical interviews. Thus, we have a pre- and post-treatment textual corpus.

**Table 1:** The set of questions of the semi-structured interviews, at baseline and at follow-up at 6 months, of current smokers who attended the Smoking Cessation Unit of the Clinic Hospital, Santiago de Compostela, Spain, 2017–2020 (N=116)

*Baseline*	*Follow-up at 6 months*
How are you? Are you ready to quit smoking? What are the reasons why you want to quit tobacco? What do you think about tobacco? What things make you smoke? How badly do you want to quit smoking? Do you see yourself able to quit smoking? Have you made any previous quit attempts? Have you used previous treatments? Do you want to add something else about tobacco?	How are you at the present time? Have you quit smoking? Have you taken the prescribed treatment? How are you in strength at the present time? What is your mood? What do you think about tobacco? Do you want to add more about smoking?

*Content analysis phase*

This phase comprised two components: 1) Qualitative analysis, and 2) Quantitative analysis.

Qualitative analysis

After reading the textual corpus at baseline and at follow-up at 6 months, the researchers established different codes or categories that defined the meaning of each of the segments of the text, generating a list of qualities of the smoker. These codes were smoking, dependence, motivation, emotionality, anger, and satisfaction. Subsequently, each code was assigned to a fragment of the text (open coding) (Table 2). These categories could then be linked through a process of identifying relationships contrasting existing similarities and differences, resulting in diagrams or networks between codes that allowed us to establish relationships and formulate the grounded theory. In other words, it was about formulating a hypothesis based on smoking before and after treatment.

**Table 2:** Codes or categories and their definitions, used in the study, Tobacco Cessation Unit, Santiago de Compostela, Spain, 2017–2020 (N=116)

*Codes*	*Definitions*
**Smoking**	Tobacco consumption intensity
**Motivation**	Patient attitude to quit smoking
**Dependence**	Manifestations associated with the need to consume tobacco
**Abstinence**	Having been previously smoking-free for more than 6 months in the case of pre-treatment, or smoking-free for more than 6 months after therapeutic intervention
**Emotionality**	Expression of emotions and feelings
**Anger**	Disgust with smoking
**Satisfaction**	Feeling of well-being when a wish has been fulfilled

Quantitative analysis

The frequency and percentage of words in the textual corpus were calculated, excluding words that did not contribute semantic value, transforming words, unifying verb tenses, and eliminating plurals. This process is called lemmatization.

### Description of the tobacco cessation intervention protocol

The smoking cessation intervention integrated into daily clinical practice consisted of a program that includes a face-to-face consultation by a therapist and nursing staff trained and accredited in smoking cessation. The criteria for inclusion in the Smoking Cessation Unit of Santiago de Compostela were: 1) Having made a previous unsuccessful quit attempt with psychological counseling and pharmacological treatment; and 2) Current comorbidities or situations that make smoking treatment more difficult and require a more intensive intervention (psychiatric pathology, heart disease, use of other drugs, or pregnancy).

In this consultation, a diagnostic approach is made to the smoker based on a set of tests applied in clinical practice. Questionnaires (smoking degree, analysis of previous quit attempts, evaluation of motivation, self-efficacy, and nicotine dependence) and complementary tests (spirometry and co-oximetry) are carried out. After that, individual cognitive and behavioral treatment is provided (thought stopping, distraction, self-instructions, self-control, problem-solving, reinforcement, cognitive restructuring, and systematic desensitization), as well as pharmacological treatment: cytisine, nicotine replacement therapy (NRT), varenicline, and bupropion. Follow-up visits were conducted at 6 months in which tobacco consumption was quantified, abstinence and its symptoms were verified, and the side effects of the treatment and the difficulties or barriers presented by the smoker during the cessation process were assessed.

### Data analysis

A descriptive analysis of the sample was performed using the IBM SPSS version 22 program. Categorical variables were analyzed by means of contingency tables, expressing the values in frequencies and percentages. Continuous variables were analyzed using means and standard deviations.

For the quantitative analysis of the textual corpus, WORDSTAT 7 Version 7.1.20 software was used to determine the frequency and percentage of words and phrases before and after the smoking cessation intervention. The qualitative analysis of the textual corpus was supported by an integrated package of QDA MINER version 4.1.33 and WORDSTAT 7.

## RESULTS

### Descriptive characteristics of the sample

**Figure 1:**
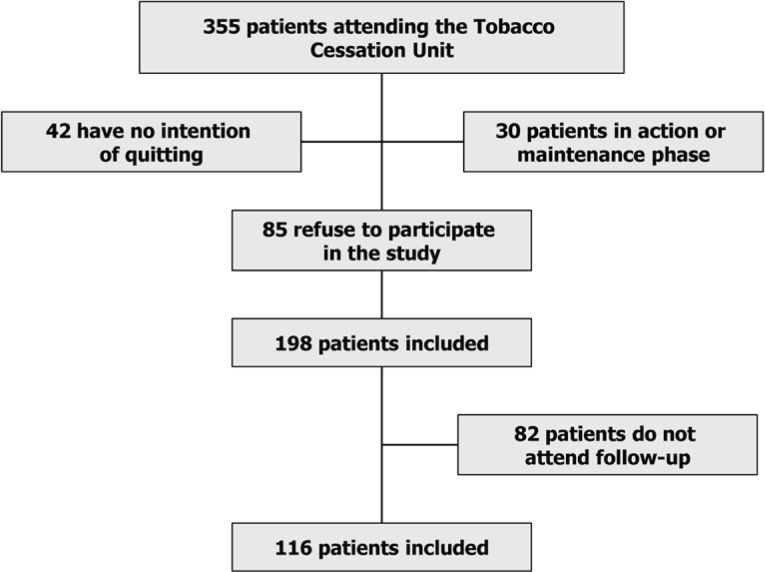
Flow-chart of patient selection

A total of 116 patients [66 males (56.9%); mean age 55.6 ± 10.6 years] were included. Figure 1 shows the flowchart of patient selection; 76.7% of patients had a basic education level, and 30% were pensioners or unemployed. Current consumption was 17.8 ± 10.7 cigarettes/day, and cumulative consumption was 45.5 ± 2.0 packs/year. The mean value of motivation calculated on the visual analog scale of motivation (VAS) was 8.3 ± 2.0. In 81% of the patients, the time until they smoked their first cigarette was less than 30 minutes. The mean value of the Fagerström test was 5.7 ± 1.6 and exhaled carbon monoxide was 13.6 ± 8 ppm. The mean Glover-Nilsson test score of the patients in the sample was 12.7 ± 7.5. All patients received psychological counseling based on cognitive and cognitive-behavioral strategies. The mean number of visits was 2.70 ± 2.35, with a minimum value of 2 and a maximum value of 8 follow-up visits. The intervention format was individualized and face-to-face. The duration of the interventions was 20 minutes for the baseline visit and 10 minutes for the follow-up visits. Fifty percent of smokers received varenicline, 35% nicotine replacement therapy, 10.3% bupropion, and 4.7% cytisine. The mean duration of drug treatment (NRT, varenicline, bupropion, and cytisine) was 40.3 ± 12.0 days. Abstinence at 3 months was 42%, and 29% at 6 months.

### Quantitative content analysis

After analysis of the textual corpus of the 116 patients in the baseline interview, the most frequent word was ‘smoking’ (871 occasions; 4.3% of the total), followed by ‘have’ (376; 1.8%), ‘want’ (224; 1.1%), ‘tobacco’ (173; 0.9%), ‘earn’ (126; 0.6%) ‘cigarette’ (120; 0.6%), ‘try’ (116; 0.57%), ‘anxiety’ (97; 0.5%), ‘force’ (80; 0.4%) and ‘able’ (74; 0.4%). In other words, the smoker’s baseline smoking history before developing the smoking cessation intervention revolves around smoking and tobacco dependence, his or her intention to quit smoking, including the reasons for trying to quit, and the obligation or commitment to do so. However, they express obstacles or barriers (‘anxiety’, ‘able’).

The most frequently repeated phrases in the initial interview reflected the predisposition to quit smoking: ‘wanting to quit’ (307 occasions), ‘being able to quit’ and having the strength to quit (104 occasions), and ‘having to smoke’, ‘being afraid to quit’, ‘having tobacco’ and ‘needing tobacco’ (35 occasions). The 13 most frequent phrases were grouped by theme: 1) referring to the intention to quit smoking, ‘wanting to quit smoking’ (56%); 2) those reflecting commitment, self-efficacy, and reasons for quitting ‘be able to quit’, ‘have the strength to quit’, ‘try hard to quit’ and ‘they will all win’ (22.4%); 3) past experiences in quitting ‘I tried to quit’ (11.86%); and 4) others reflecting exclusively nicotine dependence ‘I have to smoke’ (8.9%).

After the intervention, six months after the start of the smoking cessation program, in the analysis of the textual corpus of the 116 patients, the most frequent word was ‘smoking’ (258 times; 4.1%), ‘have’ (82;1.3%), ‘tobacco’ (58; 0.9%), ‘varenicline’ (37; 0.6%), ‘craving’ (32; 0.5%), ‘gain’ (32; 0.5%), ‘cigarette’ (31; 0.5%), ‘took’ (29; 0.5%), ‘nicotine’ (25; 0.4%) and ‘reduce’ (25; 0.4%). Words related to smoking (tobacco, cigarette, and smoking) were the most frequent, with a discrete reduction of 0.21% with respect to the baseline account (5.70% vs 5.49%). Words related to actions and attitudes of the patient in smoking cessation (‘reduce’, ‘I took’) (54) and pharmacological treatments used for nicotine dependence (‘varenicline’ and ‘nicotine’) were introduced (62).

The most frequently repeated phrases after the intervention were: ‘I felt like smoking’ (25 occasions) and ‘I have been without smoking’ (22), ‘nicotine gum’ (15), ‘I quit smoking’ (15), ‘I was without smoking’ (11), and ‘I started smoking again’ (11).

The 13 most frequent phrases appeared 143 times. Of these, 45 were related to abstinence and satisfaction with smoking cessation (31.5%); 21.7% (31/143) are related to pharmacological treatment (21.7%) (‘I took the pills’, ‘I was on varenicline’, ‘I was on nicotine patches’, ‘I use nicotine gum,’ and to a less extent, to experiences and perceptions of the smoking cessation process [‘I was without smoking’ (11 times), ‘I started smoking again’ (11), and ‘I tried to quit smoking’ (10)] (Tables 3 and 4). Therefore, the predominant phrases in the discourse at the end of the cessation program were related to abstinence and satisfaction and to the actions developed by the smoker (behavioral actions and pharmacological treatment).

**Table 3:** Quantitative content analysis of the textual corpus of the narratives of the smokers, consisting of determining the words and their frequency before and after the intervention, Tobacco Cessation Unit, Santiago de Compostela, Spain, 2017–2020 (N=116)

*Ten most frequent words at baseline*	*n*	*Ten most frequent words after the intervention*	*n*
Smoke	871	Smoke	258
Have	376	Have	82
Want	224	Tobacco	58
Tobacco	173	Varenicline	37
Gain	126	Anxiety	32
Cigarette	120	Gain	32
Attempt	116	Cigarette	31
Anxiety	97	Took	29
Force	80	Nicotine	25
Able	74	Reduce	25

**Table 4:** Quantitative content analysis of the textual corpus of the narratives of the smokers, consisting of determining the phrases and their frequency before and after the intervention, Tobacco Cessation Unit, Santiago de Compostela, Spain, 2017–2020 (N=116)

*Ten most frequent phrases at baseline*	*n*	*Ten most frequent phrases after the intervention*	*n*
Want to quit smoking	307	I wanna smoke	25
Being able to quit smoking	54	I have not smoked	22
Have the strength to quit smoking	50	Nicotine gum	15
Have to smoke	22	I am without smoking	15
Experience with tobacco	19	I was without smoking	11
Smoking again	18	I went back to smoking	11
I tried to quit smoking	18	I tried to quit smoking	10
I’ve been months without smoking	14	I was on nicotine patches	9
Afraid to leave	14	I was on varenicline	7
Make an effort to quit smoking	12	I want to stop smoking	6

### Qualitative content analysis

**Figure 2:**
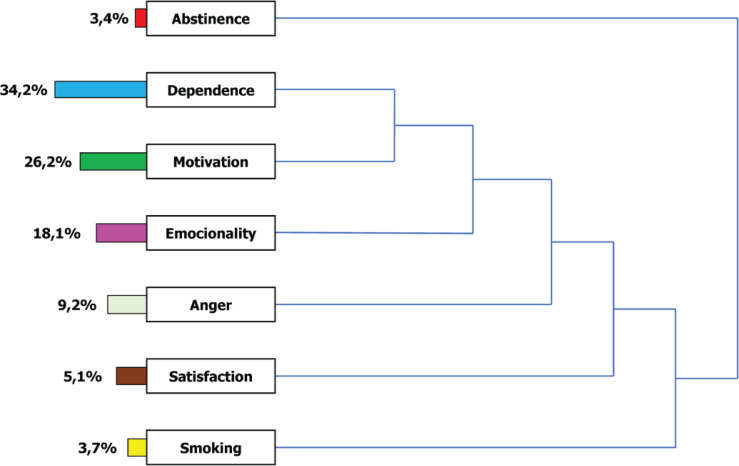
List of codes or categories and their distribution in percentages (%) present in the textual corpus of the baseline interview (N=116)

In the baseline interview, after the open coding process, the most frequent categories were dependence (394 times; 34.2%), motivation (301; 26.2%), emotionality (208; 18.1%), anger (106; 9.2%), satisfaction (59; 5.1%), and smoking (43; 3.7%). The abstinence code was the least frequent (39; 3.4%) (Figure 2).

The axial coding process allows the relationship between the different categories or codes. The code dependence was related to the code motivation and both to emotionality. All of these were associated with anger but also with satisfaction because of the support received in the smoking unit. In turn, all these codes were associated with smoking and influenced the possibility of quitting smoking (Figure 2). We thus formulated a theoretical hypothesis: smokers are subjects with thoughts that translate their nicotine dependence and their motivation to quit. In an intense emotional environment, they were torn between failing and succeeding in quitting.

**Figure 3:**
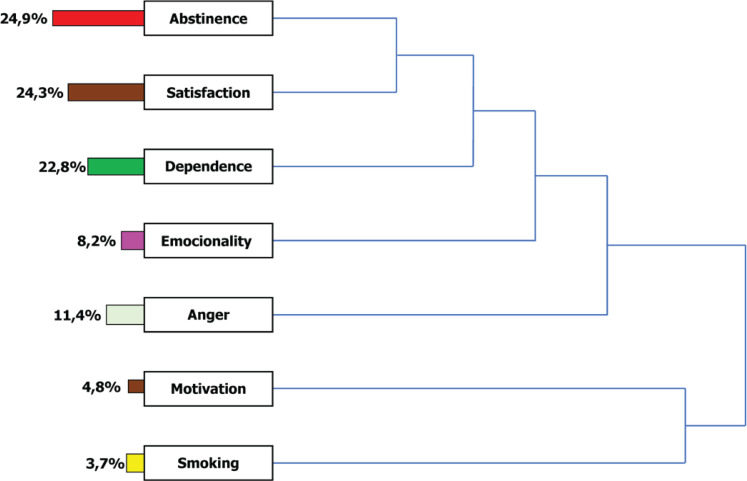
List of codes or categories and their distribution in percentages (%) present in the textual corpus of post-intervention interviews (N=116)

After the intervention, the distribution of codes was as follows: abstinence (94 times; 24.9%), satisfaction (92; 24.3%), dependence (22.8%; 86%), anger (43; 11.4%), emotionality (31; 8.2%), motivation (18; 4.8%), and smoking (14; 3.7%) (Figure 3).

In the axial coding process, the codes abstinence and satisfaction were associated with each other, and, in turn, both were associated with nicotine dependence, which was related to emotionality. The code anger was associated with all of them, and, in turn, all of them are related to motivation and smoking. In other words, the abstinence achieved by the smoker produces satisfaction and impacts nicotine dependence. All these changes act on the smoker’s sensitivity to positive and negative emotions. All these categories influence anger and determine motivation and smoking (Figure 3).

### Effects of the smoking cessation intervention on the smoker’s narrative

**Figure 4:**
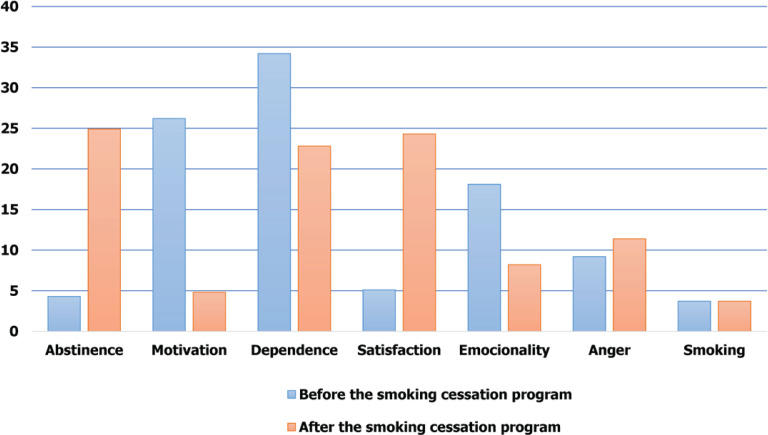
Effect of smoking cessation intervention on the percentage distribution of codes in the textual corpus of the narratives of smokers who were included in the study, at baseline and at 6 months after the cessation intervention (N=116)

A comparison of the textual corpus before and after the intervention has revealed, with regard to qualitative analysis, that smoking cessation has an effect on the distribution of the categories. Categories of dependence (11.4%), emotionality (9.9%), and motivation (21.4%) were reduced after the intervention, while there was an increase in anger (2.2%), satisfaction (19.2%), abstinence (21.5%) and smoking (21.4%) (Figure 4).

## DISCUSSION

In our study, we first found high nicotine dependence and a high degree of smoking according to the quantitative narrative analysis of current smokers who intend to quit smoking (the most frequent words were ‘smoking’, ‘tobacco’, and ‘cigarette’) and the qualitative analysis also (the code dependence is the most repeated). These results are confirmed by quantitative analysis after administering dependence questionnaires used in routine clinical practice and correlating with those from another cohort in our clinical setting characterized by highly dependent smoking populations^17,18^. This is attributed to the criteria that exist in our country to be admitted to a specialized smoking care program^19^. In addition, this is a population that is highly motivated to quit smoking. The phrase most frequently repeated by smokers at the baseline visit was ‘wanting to quit smoking’, which appeared on 307 occasions and represents 56% of the thirteen most frequent phrases. Also, in more than 75% of the textual corpus, phrases related to motivation and commitment to quit smoking were present. In the qualitative analysis, the motivation category was the second most frequent (26.2%).

These results coincide with those obtained in the descriptive analysis of the cohort, presenting a mean value of motivation of 8.3 ± 2.0 measured through the visual analog scale. In a recent content analysis study, motivation, understood as the desire to quit smoking, was present in 83.3% of the smoker’s narrative^14^. Through the axial coding process of qualitative analysis, the two most frequent codes were dependence and motivation, which are closely related to each other. Thus, a smoker with the intention of quitting tobacco is torn between the reasons for making the decision to quit and the difficulties involved in carrying it out, expressed by the need for nicotine^20^.

The second notable finding in the smoker’s baseline textual corpus is that the emotionality code, defined as sensitivity to positive and negative emotions (anxiety, stress, personal problems, etc.), was the third most frequent code. This result is justified by the profile of the smoker analyzed in this cohort (dependent and with a high degree of smoking), which is characterized by emotional dysregulation, an aspect that is associated with a slower reduction of abstinence symptoms and a greater possibility of relapse and, therefore, of failure^21^. This alteration in the regulation of emotions was associated in our study with nicotine dependence, attributes that are closely linked and confirm previously reported results^22-24^. This finding could be explained by the connectivity between areas of reward (dependence) and emotions in the central nervous system. In cohorts of smokers with greater sensitivity to negative emotions such as anxiety, functional brain imaging studies show greater activation of regions such as the striatal nucleus (reward area) and those related to the control of these emotions^25^. Other attributes present at the baseline interview, such as anger or satisfaction, were detected in a lower percentage because the smoker who proposes a quit attempt is dissatisfied with his smoking behavior and exhibits a lower degree of anger since he will probably receive support.

Finally, it is also worthy to mention the effect of the smoking cessation intervention on the smoker’s report. This intervention is based on a first visit where psychological counseling is conducted, a drug for nicotine dependence is administered and monitored over a year follow-up period, and meetings are held on a monthly basis. In the quantitative analysis, we observed a predominance of words related to smoking. However, in the analysis of the frequency of phrases, the most frequent were related to the manifestation of abstinence and satisfaction (31.4%). In the qualitative analysis, the most frequent attributes after the intervention were satisfaction and abstinence (identified in more than 24% of the textual corpus each). These results were confirmed after conducting descriptive statistics (abstinence figures were 29% at six months). Both abstinence and satisfaction were two qualities that increased 21.5% and 21.4% compared to the baseline visit. Struik et al.^14^ observed a predominance of the code ‘success of the intervention’ in 57% of the texts three months after the intervention. These results corroborate previous studies that achieve abstinence figures between 30–50%^1-4^.

It has also been observed that smoking cessation increases the degree of patient satisfaction^26^. However, the usual clinical research ignores the impact of this type of intervention on other qualities of the smoker. Thus, in the post-intervention quantitative analysis, we observed the appearance of new characteristics, such as the smoker’s experiences during the smoking cessation process or the assessment of the pharmacological treatment used^13-15^. In the postintervention qualitative analysis, we observed an 11.4% reduction in the dependence category and a 9.9% reduction in emotionality. After axial coding, the reduction in dependence was associated with a reduction in emotionality. The absence of nicotine exposure and drugs to treat dependence would decrease sensitivity to emotions. Thus, there are studies that relate the progressive reduction of negative emotions (anxiety, stress, depression) with smoking cessation^23,27-30^.

In this study, we present a new methodological approach to have better insight into different patterns of the smokers ready to quit smoking, including emotionality, satisfaction, motivation, or anger, as well as their variation during the smoking cessation process, which allows a better approach to smoking by the clinician. All this will make it possible to design personalized strategies for smoking cessation and to incorporate diagnostic tests that analyze these aspects of smoking cessation care. It is one of the first studies that use content analysis as a technique that makes it possible to evaluate all these qualities that are difficult to quantify.

### Limitations

Study limitations include the relatively small number of patients treated at a single center and that only the most nicotine-dependent smokers were included. The duration and intensity of the interventions were heterogeneous, although all received a minimum of 3–4 visits lasting at least 15 minutes. Other limitations of the study are the sampling approach and the limited generalizability to other countries. We have not analyzed the smoking narrative by sex, by smoking cessation intervention, or by treatment failure.

The study of the smoker’s narrative of who wants to quit smoking could allow us to personalize the intervention, increasing its efficacy. When nicotine dependence predominates in the narratives, it would be necessary to increase the dose or duration of drugs, whereas other groups with greater sensitivity to external stimuli and emotions would benefit from psychological intervention, and it should be reinforced. For those patients focused on less self-efficacy and motivation, the motivational sphere should be treated (deepening the motivational interview, intervention with families, or changing to a group format).

## CONCLUSIONS

The study of the smoker’s narrative in a smoking cessation unit through content analysis makes it possible to identify unknown aspects of the smoker and their variation as a consequence of smoking cessation.

## Data Availability

The data supporting this research are available from the authors on reasonable request.
